# Green space and the health of the older adult during pandemics: a narrative review on the experience of COVID-19

**DOI:** 10.3389/fpubh.2023.1218091

**Published:** 2023-07-31

**Authors:** Nasibeh Tabrizi, Azadeh Lak, S. Mohammad Reza Moussavi.A

**Affiliations:** Faculty of Architecture and Urban Planning, Shahid Beheshti University, Tehran, Iran

**Keywords:** older adults, COVID-19, green space, health, narrative review

## Abstract

**Introduction:**

Aging is an inevitable process that leads to changes in various dimensions of older adult life, including physical, psychological, and social aspects. Unfortunately, older adults are more susceptible to health problems caused by adverse experiences such as the Corona outbreak.

**Aim:**

The current study examines the lived experience of older adults in facing the conditions of the Corona epidemic to see how green spaces at various scales can influence the physical and mental health of this group.

**Method:**

Relevant articles published, from 2019 to February 17, 2023, were searched using in the Scopus and Web of Science databases. Eligible studies published in English and all studies passed a quality evaluation.

**Result:**

In the final search, 40 articles were selected and analyzed. The majority of studies conducted during the pandemic categorized the impact of green spaces on the health of older adults into three main categories: Place-based attribute, Process, and Function.

**Conclusion:**

The findings of this study demonstrated that people were using private green spaces (gardens, balconies, etc.), small local green public spaces, sitting and gathering spaces in the neighborhood, nearby open spaces, and urban green-blue spaces throughout the epidemic era. They visited green spaces outside the city and urban areas, including urban gardens, agricultural areas, forestlands, and pastures. In this research, we investigated the characteristics of these spaces and classified them into four physical categories: urban landscape, land use, activity, movement, and accessibility. The results showed that exposure to nature or green space improved physical and mental health and increased attention and decision-making quality in older people. We have proposed design implications recommendations for crises to improve safety, security, and social capital by increasing the safe access of older adults to diverse and high-quality green spaces on different scales, which will ultimately enhance the physical and mental health of people in different age groups.

## 1. Introduction

The health issues caused by the COVID-19 pandemic have had severe repercussions for urban areas, including detrimental impacts on urban health, society, and the economy (1). COVID-19 has spread to at least 180 countries around the world and in less than a month, caused 95,000 confirmed cases and almost 3,200 deaths [Chen et al., 2020, as cited in Adjie and Bahari (2)]. As a result, governments have implemented various lockdown (official order to control the movement of people or vehicles) (3). Policies included, restrictions on attendance at public events and gatherings, restrictions on the use of public transportation, and school closures. Administrations also implemented and created public awareness campaigns about COVID-19 (4). Therefore, many developments have occurred in various socio-economic, cultural, and physical dimensions of the living environment in urban societies, especially for vulnerable groups such as children and older adults.

Access to urban green spaces improves people's quality of life in cities as it allows for the creation of jobs and food, the promotion of biodiversity, urban heat mitigation, and the creation of health advantages (5). Increasing or improving green spaces in public places can have a good impact on all demographic groups, particularly vulnerable groups such as older adults and children (6). While older adults need to live in cities for better social support (7), access to green spaces and usage of health benefits is particularly vital for this population (8). Access to these spaces helps to address public health issues such as obesity, cardiovascular impacts, mental health, and wellbeing (6). Studies have shown that coronavirus has had a stronger impact on most people over 60 years old because the case fatality rate (CFR), which is the ratio of death to infection, is higher in people over 60 years old (4.5%) than in people who are 60 years old or younger (1.4%) (9). The World Health Organization has recommended home lockdown and avoidance of contact with others for this age group, which undoubtedly impacts various aspects of their health, particularly their mental wellbeing (10). In addition, the increase in mental health issues caused by the spread of COVID-19 has led to the disconnection and reduction of social connection for older adults, which exposes them to depression and anxiety and increases their vulnerability (11). Moreover, self-isolation and loneliness can increase the risk of suicide among the older adult population (12). According to Wang and Li (13), the COVID-19 outbreak, as the most prevalent disease, has been the major culprit of moderate-to-severe depression symptoms for 16.5% of urban residents and accounts for 28.8% of moderate-to-severe anxiety symptoms and 8.1% of moderate-to-severe stress symptoms over time.

The COVID-19 crisis has highlighted the vulnerability of the older adult population, particularly in terms of their physical (14) and mental health (15). The pandemic has caused a range of concerns for this demographic including anxiety, depression [(11); Huang and Zhao, 2020; Wang et al., 2020; Zhu et al., 2020, as cited in Dzhambov et al. (16)], uncertainty about the future, limited physical activity, and changes in diet due to fear of going shopping and limited access to health services. As a result, the pandemic's impact on the social, economic (17) environmental, and health aspects of older adults' lives has been significant, and there has been a need to find solutions to mitigate its adverse effects. Urban design has been identified as a potential solution to alleviate the negative impact of the pandemic on older adults. Physical-spatial features of living environments have an impact on the wellbeing of older adults during pandemics and epidemics. Urban green spaces positively affect their health and access to green spaces during lockdown reduces stress, anxiety, and physical health issues (6).

Prior to COVID-19, research consistently shows nature exposure improves mental health and wellbeing. Urban green spaces, water bodies, private gardens, and visual experiences are linked to these benefits. Nature connections also reduce the risk of psychiatric disorders [Bratman et al., 2019; Enge-mann et al., 2019; Tost et al., 2019; De Bell et al., 2020; Jarvis et al., 2021; White et al., 2020; (6) as cited in Nigg et al. (18)]. COVID-19 has emphasized the importance of engagement with green spaces to cope with the stress of the virus' danger and the physical constraints imposed in response (19). A study conducted by Berdejo-Espinola et al. (20), found that people with access to green spaces during lockdown experienced a significant decrease in stress (59%), anxiety (55%), and self-reported physical health symptoms (57%). In addition, other urban design features such as pedestrian qualities, a pleasant environment, and accessible parks have also been found to have a significant impact on health levels of older adults.

According to a study by Zhu and Xu (21), individuals over the age of 60 reported higher levels of life satisfaction during lockdown compared to other age groups. These individuals were able to handle difficult situations more effectively due to their flexibility in daily activities and plans. Older individuals with physical limitations and limited mobility had access to local public spaces for physical activity in their neighborhood. During lockdown, older adults were able to adapt to the situation by using social networks, engaging in mental and physical activities, gardening, changing their daily routines, and adjusting to the circumstances (22). Therefore, when designing and planning residential areas, the specific physical needs of older individuals should be considered.

The COVID-19 pandemic has put the physical and mental health of older adults at risk. However, research has consistently emphasized the importance of green space in improving the health of this vulnerable population (23). However, studies reveal that connected urban green spaces expand people's choices, which increases the spread of the epidemic and makes older adults more vulnerable (24, 25). But numerous studies have shown that the connection of older adults with green spaces on various scales and accessible social distance lowers the physical and psychological symptoms of lockdown, such as desperation, anxiety, mood swings, etc (5, 26, 27). Given the negative social and individual effects of infectious disease on older adults, it is necessary to identify how the physical-spatial factors of their residence can improve the health of older adults in their living environment and neighborhood and identify what role green space plays at different scales. Understanding how measures like lockdown can prevent irreparable consequences and experiences caused by loneliness is crucial.

**Figure 1:**
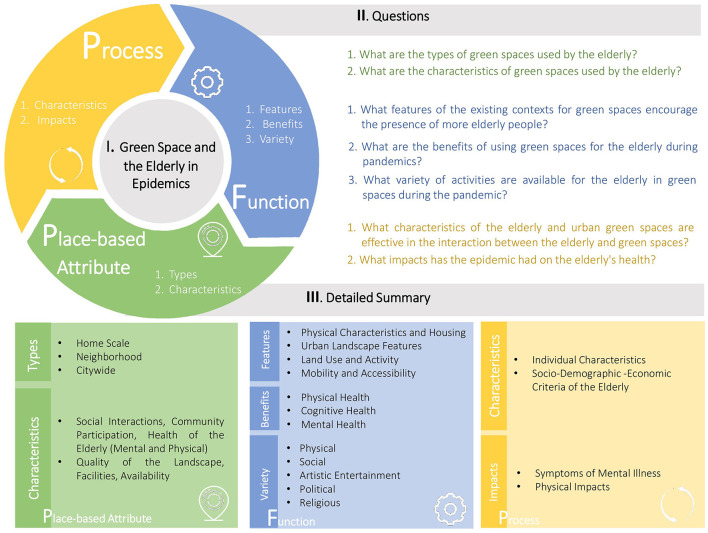
Conceptual framework of green space and older adults in epidemics.

We try to gain insight into various countries and their experiences. For example, In Ireland, the term “cocooning” emerged, describing the act of staying indoors and isolating oneself from perceived risks rather than going outside. As a result, we employed a qualitative study method to delve deeper into these occurrences and better understand the situation (28). This narrative review study aims to provide a conceptual framework for planning aging in place during the pandemic, focusing on physical-spatial factors. To accomplish this, we reviewed existing literature that discussed the various impacts of the epidemic on this relationship. Based on our findings, we developed a conceptual framework (Figure 1) that aligns with our research question. The following questions represent the focus of our research:

- 3.1. What impact has the epidemic had on older adults' health?
- 3.2. What are the benefits of using green spaces for older adults during pandemics?
- 3.3. What characteristics of older adults and urban green spaces are effective in the interaction between older adults and green spaces?
- 3.4. What variety of activities are available for older adults in green spaces during the pandemic?
- 3.5. What features of the existing contexts for green spaces encourage the presence of older people?
- 3.6. What are the characteristics of green spaces used by older adults?
- 3.7. What are the types of green spaces used by older adults?

Several articles have explored the utilization of green spaces prior-to and after the COVID-19 pandemic. However, there has been a scarcity of studies focusing on specific population segments, particularly older adults. Although some studies have examined the effect of green spaces on older adults, such as Wu et al. (29), Ali et al. (30), Guida and Carpentieri (31), and Rantanen et al. (32) studies, they have not comprehensively addressed the different aspects of this effect during the epidemic. Therefore, there was a need to examine the specific design implications for green spaces catering to this particular age group.

To gain international insights, the focus was placed on examining various countries, including both developed and undeveloped nations. By considering a diverse range of countries, a more comprehensive understanding of the impact of green spaces on different populations could be achieved. This approach allows for a broader perspective on how green spaces are utilized and their design requirements across different socio-economic contexts.

## 2. Literature search methodology

While this review is narrative in nature, a systematic search was conducted via Scopus and Web of Science (33) from 2019 to February 17, 2023 in order to ensure that relevant studies were not overlooked (33). The keywords for the search were “urban green space,” “coronavirus,” and “older adult people.”

**Figure 2:**
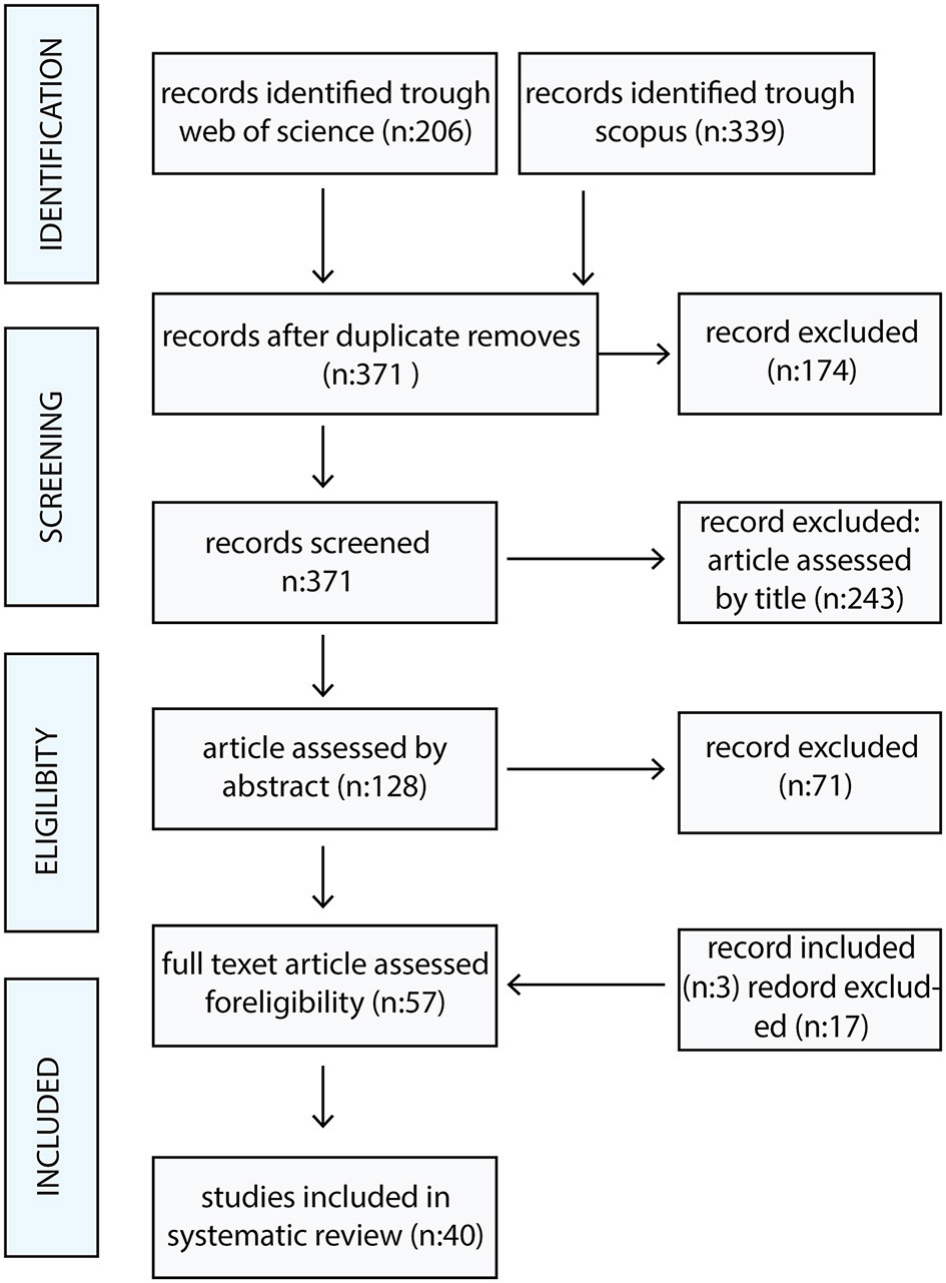
Flowchart of article selection.

In the initial search, 339 and 206 articles were identified; after removing duplicates and studies with only an abstract, 371 articles remained. Finally, two inclusion criteria were used to select articles: (a) articles should be written in English; (b) they should be on the relationship between the features of the built environment and the physical health of older adults. A total of 40 articles were ultimately chosen and evaluated after examining the titles and abstracts of 128 articles (Figure 2). Finally, a conceptual framework with three categories was developed: Place-based attribute, Process, and Function.

## 3. Results

### 3.1. What impact has the epidemic had on older adults' health?

The unequal distribution of COVID-19 and the uncertainty associated with this disease have harmed the health of older adults on both an individual and communal level (34). Oliveira et al. (35) have found that the degree of physical activity and lifestyle during the pandemic have had a significant impact on older adults. A considerable fall in physical activity levels throughout the lockdown has negatively influenced the level of physical fitness and increased sedentary lifestyles factors that have been directly associated with increasing weakness in this group.

Studies show that the mental health status of older adults has been negatively impacted during the pandemic. Some preventive measures that have been taken to increase the resilience of older adults seem to change their living conditions during lockdown and help them deal with self-isolation, social distancing, and stress disorders. However, in their study, Perlman and Peplau (36) showed an increase in aging symptoms in people due to a decrease in immunity and a rise in anxiety during this period. Loneliness or the lack of social relationships was among the issues that older adults experienced more than other people due to being in a high-risk group.

This group was also affected by the adherence to lockdown rules and the lack of communication with the outside world, the emotional loneliness brought on by the absence of an intimate relationship or an emotional dependency [Weiss, 1974, as cited in Gaaloul et al. (37)], and the social loneliness caused by the absence of communication with a larger group of contacts. Van Tilburg et al. (38) asserted that many older individuals adopted social media as a substitute for in-person communication during the epidemic because of the decline in social connections. According to Pouso et al. (26), there was no significant difference in the level of depression between people with partial restrictions and people without restrictions to access public open spaces and green spaces. This result showed the importance of contact with nature to maintain mental health and emphasized the quality of being in green space rather than its quantity. Despite the time limit imposed by authorities, the use of green spaces has a great impact on mental health (26) and the physical health of older adults (39).

The sense of isolation among older adults can also be attributed to individual factors. Research has shown that age greatly impacts access to services and amenities, as well as participation in cultural and leisure activities. Additionally, factors such as retirement and marital status may contribute to feelings of loneliness and isolation (40). Despite the effect of physical distancing policies on social isolation, such effects are only seen in married people or those who are not in touch with others through social networks (38).

In the various reviews, some policies need to be established to create friendly communities for older adults. In this context, places far from the community should be examined, people far from social activities should be studied, and policies at the neighborhood scale to increase social participation should be considered [(38); Table 1] shows the effects of Corona on the health of older adults.

**Table 1:** The effects of coronaviruses on the health of older adults (mental and physical; source: Authors).

**Characteristics**		**The number of repetitions**	**References**
**Symptoms of mental illness**	Anxiety	11	(9, 10, 15, 16, 24, 26, 27, 38, 41–43)
	Depression and frustration	9	(9, 10, 15, 16, 24, 26, 27, 38, 41)
	Fear	6	(16, 24, 27, 38, 44)
	Social relationships of the individual	5	(32, 44–47)
	The prevalence of the disease	1	(48)
	Lack of desire and pleasure in doing things	1	(24)
	Increasing of spending time at home	2	(16, 46)
	Bad temper	1	(27)
	Sorrow	1	(38)
	Weak concentration	1	(27)
	Changes in diet	1	(16)
	Suicide (especially in nursing homes)	1	(9)
	Homesick	1	(38)
	Decreased cognitive ability in old age	1	(41)
	Decreased mental activity	1	(44)
	Absurdity	1	(38)
	The feeling of crime	1	(24)
	Personality characteristics	1	(41)
	Returning to mental habits (alcohol, drugs, cigarettes…)	2	(9, 43)
	Inability to control worry	2	(24, 27)
	Quality in sleep	3	(10, 16, 27)
	Tiredness	2	(16, 27)
	The amount of physical activity inside and outside the home	5	(39, 44, 47, 49, 50)
	Increase in body mass index	1	(10)
	Height and weight	1	(51)
	Reduce physical activity	3	(38, 42, 51)
	Reducing the percentage of positive tests	2	(15, 52)
	Deterioration of diet	1	(44)
	Increasing the amount of disability	1	(53)

### 3.2. What are the benefits of using green spaces for older adults during pandemics?

Numerous studies have demonstrated that exposure to natural environments offers extensive physical and psychological benefits (18). These effects have been observed in various study designs, including experimental, ecological, cross-sectional, and longitudinal studies. It is important to note that the definition of “natural environment” varies among these studies and encompasses green spaces, parks, nature, outdoor environments, and more. Specifically, exposure to green spaces serves three key functions: reducing harm (e.g., minimizing air, noise, and heat pollution), restoring capabilities (e.g., physiological restoration and improvement), and fostering capabilities (e.g., promoting physical activity and facilitating social cohesion). Despite the diversity in study designs and definitions of exposure, we have compiled relevant evidence to establish the positive impact of outdoor environments (natural or built environment) on various health domains, as summarized in Table 2.

**Table 2:** The positive impact of external environments (natural/constructed environment) on different areas of health [source: Levinger et al. (1)].

**Physical health**	**Cognitive health**	**Mental health**
• Increase physical activity • Increase walking • Increase participation in casual/recreational activities • Increasing adherence to recommended guidelines in physical activity • Improving physical disorders and functional limitations • Reducing mortality from various diseases • Physiological benefits (e.g., increased immune system function)	• Improve focus and attention • Overcoming mental fatigue • Improving global decision-making processes	• Improves mood • Reducing stress • Increasing social connections

During lockdown, it is possible to enhance the mental and behavioral health of older adults by considering their personality traits, such as higher intelligence, emotional stability, and extroversion, while also promoting public communication [Killingsworth and Gilbert, 2010; Tost et al., 2019 as cited in Nigg et al. (18)]. Access to outdoor recreational facilities and nearby parks, as well as increased physical activities, can contribute to maintaining physical and mental wellbeing and establishing safe social relations during the pandemic, as environmental-spatial characteristics play a role in these aspects (1). Urban parks don't only benefit mental and physical health but also facilitate the development of social relations within neighborhoods [Hayward and Weitzer, 1984; Lloyd et al., 2008 as cited in Addas et al. (54)]. Considering the special physical needs of older individuals, it is crucial to ensure their presence and provide thoughtful design and planning of outdoor environments in residential areas during pandemics.

In a study by Bartalucci et al. (55), the comparison of the data before and during the lockdown period showed some improvements in people's perception of nature and the sounds of the environment. During the lockdown, people paid more attention to the sounds around them. Such improvement in the sound landscape was associated with the reduction of road, air, and rail traffic (55). The study also found that older people over the age of 60 were more sensitive to traffic noise during the lockdown period, which was likely associated with understanding sounds and their natural environment. Furthermore, with the reduction of traffic noise, the quality of the soundscape improved (56). The study claims that if a person lives alone, their judgment of various aspects of their surroundings will be more positive. However, the lockdown itself can hurt human perception. Changes in people's behavior due to an epidemic like COVID-19 can have positive effects on the environment, particularly regarding the soundscape (55).

The emphasis on green space in previous reviews has been consistent. However, other forms of nature, such as blue space [e.g., rivers or lakes; Britton et al., 2020 as cited in Nigg et al. (18)], green infrastructure, which pertains to a deliberate network of open spaces, and digital nature experiences (18) have unfortunately been overlooked.

### 3.3. What characteristics of older adults and urban green spaces are effective in the interaction between older adults and green spaces?

In 40 selected articles, the focus was on factors such as the density of commercial facilities, the density of schools, access to public transportation, the density of roads, and access to green spaces, along with the environmental characteristics of built space, such as urban density and mixed language use, the health impacts of urban residents' land use, and long-term active travel behaviors.

Older adults, as a vulnerable group of people to epidemic diseases, rarely use green space due to the dread of infection (57). They are subject to the negative impacts of social isolation, dread of disease, despair (17), and depression (58). Providing green spaces in the vicinity of areas where a large number of older people live can satisfy their need for greenery, prevent social isolation, despair, and increase social interactions (20). As physical activity is closely linked to mental health, older adults' limited physical activity and lack of access to physical activities either online or at home during lockdown have damaged their health (41). Inactivity intensified by social isolation measures to combat COVID-19 can make the health conditions of older adults worse than others, resulting in sarcopenia, weakness, and cardiac abnormalities, which all can potentially lead to increased mortality issues. These problems can put them at risk of death. Efforts to develop measures related to public health and clinical interventions in physical activities are necessary during COVID-19 (49). In one research they concluded that older users of green spaces tended to spend more time in their yards than those who did not visit green spaces much [Lin et al., 2014, cited in Berdejo-Espinola et al. (20)]. This is against the assumption that those who have access to green spaces are not in the need of greenery.

This narrative review study shows that some socio- demographic factors (such as age, gender, and education) (54), and activity status of older adults, along with their racial characteristics, affect the use of urban space by older adults. A variety of factors, such as the socio-economic characteristics (59) of the place of residence, the sense of isolation and loneliness (53) and the attachment to the place, have also affected the quality of their presence in the urban space. Table 3 shows a summary of the conducted studies. Visitors' behavior in green spaces correlated with their status and occupation. Local residents who owned their own houses, had long years of housing, stable jobs, and higher education outperformed others in behavior and health perceptions when visiting green spaces (65).

**Table 3:** Individual and socio- demographic characteristics of residence of older adults (source: Authors).

**Characteristics**			**The number of repetitions**	**References**
**Individual characteristics**	Age		20	(10, 15, 16, 20, 21, 24, 26, 27, 31, 44, 46, 50–53, 55, 60–63)
	Gender		17	(10, 15, 16, 20, 21, 24, 26, 27, 41, 44, 50–52, 55, 60, 61, 63)
	Education level		13	[(10, 15, 24, 26, 27, 38, 41, 44, 50, 53, 55, 62, 64)
	Ethnicity		7	(16, 46, 52, 53, 61, 62)
**Socio-demographic -economic criteria of older adults**	**Socio-economic situation of the neighborhood**	Social support	2	(16, 64)
		Poverty	3	(52, 53, 61)
		Marital status	3	(26, 38, 62)
		Language	3	(25, 38, 53)
		Number of men and women	1	(52)
		Children	2	(26, 61)
		Number and capacity of nursing homes	1	(53)
		Family size	5	(15, 16, 26, 61, 62)
		People in need of care	2	(26, 53)
		Health insurance status	3	(52, 53, 62)
		Income	11	(10, 16, 20, 24, 26, 27, 38, 44, 46, 50, 52)
		Employment-unemployment	11	(10, 24, 26, 27, 38, 41, 52, 53, 55, 62, 64)
	**Loneliness**	The presence of pets in the house	2	(26, 27)
		Duration of visiting the green space	1	(24)
		Social isolation	4	(9, 38, 42, 44)
		Social security	1	(48)
		Living alone	5	(10, 27, 41, 53, 63)
	**Sense of belonging**	People's participation in neighborhood activities	3	(5, 46, 64)
		Duration of living in the place	1	(46)
		Sense of belonging to the neighborhood	2	(16, 64)

### 3.4. What variety of activities are available for older adults in green spaces during the pandemic?

During the COVID-19 pandemic, the requirements to observe social distancing highlight to what extent the quality of life (QOL) is related to social and physical activity in middle and old age. They also show the necessity for more mobility in the living space. During lockdown, the amount of activity and mobility of middle-aged people at home or in private outdoor spaces decreased (32). A study conducted among 60-year-old people in the early stages of social distancing in Sweden in 2020 showed that the level of satisfaction with the quality of life and feeling of loneliness did not change much, but the level of health and satisfaction with economic conditions changed, and as time went on, a greater impact on the physical and mental conditions of older adults was observed. This is because social distancing policies have either reduced or eliminated opportunities for outdoor activities such as events, artistic entertainment, social gatherings, sports classes, and political and religious meetings (32).

Urban green spaces are mainly used for various activities, such as running, walking, and outdoor sports, which depend on many interconnected variables and the type of city texture, including the construction and population density of the neighborhood, the amount of green space available, and the safety and security of traffic and crime (66). With regards to older people, men tend to exercise alone while women tend to exercise in groups. However, due to the spread of COVID-19, the absence of group activities and gyms may have led to a reduction in the time women spend walking (60). There is no doubt that the presence of large parks within residential areas has increased the amount of walking, especially for older adults, and the relationship between the distance to the park and the change in the amount of daily walking in older women is very evident (60). According to a survey, 40% of park-users are aged 60 years or older, and among this age group, 90% visit the park on a daily basis. In summary, the survey indicates that ~86% of older individuals visit a green space located within a 1-kilometer distance from their residence (30).

The study by Noszczyk et al. (67), in which 1,250 people participated, shows that people mentioned different reasons for visiting green spaces: walking outdoors (69.8%), improving general health (68.9%), access (65.7%), and the need to connect with nature (60.6%). It is interesting to note that playing sports at gyms and walking with pets were not the main reasons for residents to visit green spaces (67). More than 75% of the participants claimed that visiting green spaces had a very (42.2%) or great (34.5%) effect on reducing their stress, and only 4.3% believed that visiting green spaces was not effective in reducing their anxiety. More than 60% of the respondents expressed the need for physical activity, the possibility of spending time with family and friends, and reducing the feeling of depression as the reasons for visiting green spaces during the COVID-19 pandemic (67). In another study, the most important reason for people to visit green spaces was to improve their mental health (77.4%), followed by increasing their health and physical activity (62.8%) and the possibility of connecting with nature (52.4%). Being in green spaces to use clean air was the main reason for 32.0% of the people, while the need for gathering and social connection was only important for 6.4% of the participants (20). Visiting green spaces is primarily motivated by individual health concerns, as demonstrated by a survey conducted in another country. For example, in a survey conducted by the Department of Planning, Industry, and Environment in Australia, personal sports activities such as walking, running, fitness sports, and cycling were found to be the main reasons for using public spaces [NSW Department of Planning, Industry and Environment, 2020, as cited in Levinger et al. (1)]. Furthermore, older adults reported walking on the streets near their residences. From this, scholars emphasize the importance of “no traffic” streets for people to continue exercising with social distance (68).

### 3.5. What features of the existing contexts for green spaces encourage the presence of more older adult people?

**Figure 3:**
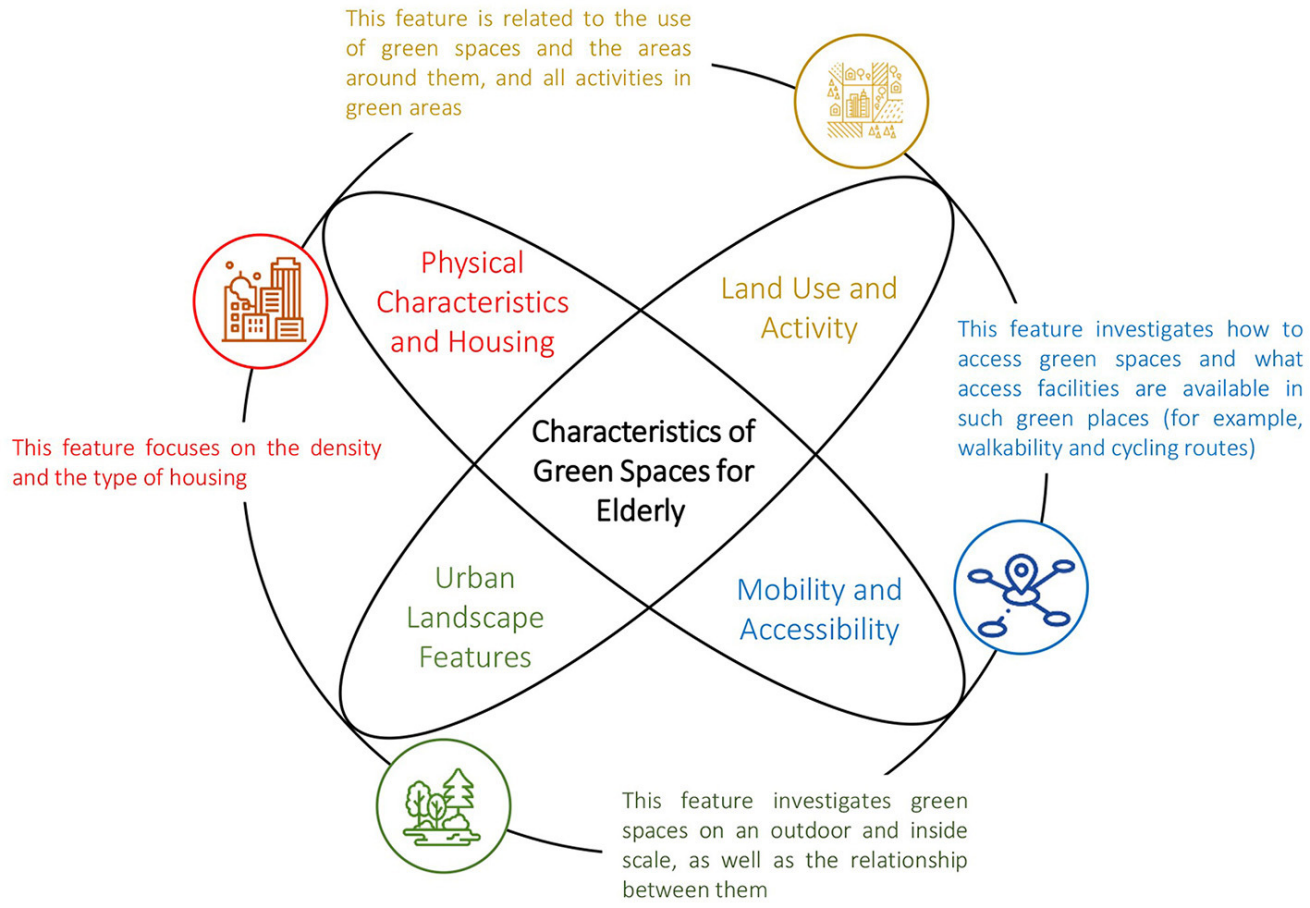
Characteristics of green spaces for older adult.

Several studies have indicated that the physical and spatial characteristics of a neighborhood can influence the attendance of older adult individuals during the COVID-19 pandemic. Factors such as the physical attributes of housing, urban landscapes, land use, and activity levels have been found to impact the quality of attendance for this population (Figure 3). Notably, the building and population density of the neighborhood, as well as the quality of corridors providing views to green spaces inside and around the neighborhood, have shown the greatest impact (Table 4). The availability of transportation options, such as vehicles, public transportation, bicycles, and access to technology in the neighborhood, including routing and augmented reality (AR) technology, elevators, sound-sensitive doors, and smart homes, have also encouraged greater attendance among older individuals. Furthermore, increased visits to urban green spaces were closely related to the distribution of green spaces around parks and neighborhoods (65, 71).

**Table 4:** Characteristics of green spaces (source: Authors).

**Characteristics**		**The number of repetitions**	**References**
**Physical** **characteristics** **and housing**	Density	7	(42, 50, 52, 55, 60, 61, 69)
	Housing type	1	(16)
	Topography	1	(31)
**Urban landscape features**	The palette of the environment	1	(15)
	Indoor greenery	3	(10, 16, 27)
	Sky landscape	1	(15)
	Artworks at home	1	(15)
	The quality of receiving light in the house and neighborhood	1	(27)
	Soundscape	2	(47, 55)
	The amount of urban green space	2	(20, 64)
	Lighting and lightening	2	(42, 47)
	Tree-lined streets	1	(45)
	Proportion of urban or rural residence	1	(24)
	A view of greenery from inside the house (outside view)	4	(15, 16, 26, 27)
**Land use and activity**	Diversity and density of uses around the green space	2	(50, 70)
	The number of green spots around the neighborhood	1	(48)
	Number of parks	1	(61)
	Brownfield	1	(48)
	Possibility of presence with pets	2	(45, 47)
	The possibility of taking children out	1	(45)
	Access to technology in the neighborhood	6	(22, 32, 44, 53, 62, 64)
	Connecting neighborhoods and green spaces with designed routes	1	(52)
	Access to home garden and fresh fruits and vegetables	1	(47)
	Access to urban agricultural lands	1	(66)
	Access to green-blue infrastructure		(47, 70)
**Mobility and accessibility**	Access to public transportation	2	(31, 42, 47, 60, 64)
	The distance to the neighborhood market	5	(69)
	Mode/type of travel (having a vehicle, public transportation, bicycle, etc.) to the green space	1	(42, 45, 47, 53, 61, 62, 70)
	Security of intersections	6	(70)
	Congestion and reduction of traffic	1	(42, 69, 70)
	Street design and pattern	3	(50, 70)
	Percentage of sidewalks	2	(42, 61)
	Bicycle route	2	(70)
	Walkability	1	(44, 47, 70)

The characteristics of the local environment in the neighborhood level play a vital role in promoting adaptive behaviors and enhancing the health and wellbeing of older adults. For instance, a study conducted in New York demonstrated that individuals living in disadvantaged neighborhoods, who faced challenges in leaving their homes due to physical disabilities, encountered similar difficulties (1). Those with functional limitations or a perception of an unsafe neighborhood had significantly lower mental health compared to those who considered their area of residence safe. This disparity in mental health could be attributed to reduced social cohesion in these neighborhoods, which exacerbates the negative effects of a lack of safety [Cramm et al., 2013; Carrapatoso et al., 2018; Choi and Matz-Costa, 2018; Bonaccorsi et al., 2020, as cited in Levinger et al. (1)].

However, the presence of greenspace alone does not guarantee access. Barriers such as inadequate transportation, connectivity infrastructure, provisions for disability, and socio-cultural issues, including harassment and violence against women, continue to persist (72). In summary, the benefits of green infrastructure depend on individuals' previous experiences with greenspace, perceived accessibility, and personal connections to the landscape [Wang et al., 2015; Roberts et al., 2019; Grilli et al., 2020; Groshong et al., 2020; Sonti et al., 2020, as cited in Maurer et al. (72)]. Additionally, socio-demographic attributes have a substantial impact on the use, attitudes, and perception of urban parks (54).

### 3.6. What are the characteristics of green spaces used by older adults?

Neglecting the quality of design in green spaces, both in terms of their qualitative and quantitative aspects, can have detrimental effects on social interactions, civic participation, community involvement, and the health of older adults [Hassen and Kaufman, 2016, as cited in Levinger et al. (1)]. Due to concerns about disease transmission, people have been avoiding public transportation, leading many cities, such as New York, to allocate open spaces and streets primarily for pedestrians and cyclists (61).

While engaging in outdoor physical activities and being exposed directly to nature have been found to have a more positive impact on health, indirect contact with green spaces, such as viewing them from a window, has been evaluated positively for maintaining the mental health of individuals during strict lockdown conditions, as observed in Spain (26).

Although the restrictions imposed during the COVID-19 pandemic may not have altered the need for urban green spaces, they have increased people's desire to visit a variety of green spaces. For instance, some individuals have traveled outside the city, while others have stayed within urban areas. Tree-lined streets and urban green spaces have been particularly sought after. As a result, the desire for trips with environmental effects has grown when access to pocket parks is not available (45). The older adult population not only requires green spaces but also tends to travel longer distances within or outside the city to access urban green areas. The green spaces utilized by older adults should be located in densely populated environments and possess important features in terms of land use (45). The availability of green pathways within the street network is one of the factors influencing older adults when selecting a location for physical activity. Other factors, including the quality of the landscape, amenities, the surrounding environment, and space availability, also play a role in people's choices. In order to enhance access to appropriate infrastructure for walking, cycling, and engaging in key activities while maintaining social distancing, the need for a variety of transportation options increases, as it not only enables older adults to participate but also contributes to societal cohesion and flexibility through increased social interactions (73).

### 3.7. What are the types of green spaces used by older adults?

The difference in the use of green spaces by older adults is associated with their physical limitations, socio-economic characteristics, and living conditions. The green space used by older adults has been studied at different scales, ranging from home green spaces, neighborhood yards, and gardens, to large urban areas (21). In a study conducted in England and Scotland in 2020, it was found that older people spent less time in green spaces during lockdown (24). The fear of infection and death caused this age group to comply with the lockdown conditions, which led to many problems for the health of this group of people. Many studies have claimed that the amount and frequency of use of green spaces by older adults is related to their health [(1, 16, 20, 22); Guida and Carpentieri, 2021]. Appendix Table 1 briefly presents the types of green spaces used by older adults during the pandemic.

Pouso et al. (26) investigated the effect of interacting with nature on depression levels using indirect access (containing the presence of both an urban and natural scene or a combination of both) and direct access (including the presence of a garden and green space and access to a terrace balcony).

Posuso et al. identified three levels of lockdown: level 1, which permits people to go out only for essential jobs, buying food and medicine, emergencies, or walking the dog (as implemented in China, Italy, or Spain); level 2, which enforces strict lockdown but allows for outdoor exercise on certain days (as implemented in France or England); and level 3, which also enforces strict lockdown but permits outdoor exercise on certain days (as implemented in France or England) (26). Also, the restriction of movement is a recommendation rather than a binding rule. This is most likely due to the high feeling of comfort from the absence of infection and the possibility of the naturalness of the space compared to balconies, compared to being in public or shared green spaces (26).

According to Pouso et al. (26), contact with nature through houseplants and balcony gardens can reduce the probability of depression and anxiety symptoms only for people in severe lockdown. As other groups leave the house more often, access to nature becomes easier for them, and in this way, stress can be reduced. The existence of private gardens and gardening spaces in patios can contribute the most to improving mental health during the pandemic as it provides access to open space and increases physical activity (26).

The diversity and abundance of plants and greenery that can be seen from windows, terraces, balconies, or in the neighborhood, as well as the addition of having houseplants or a garden, and greenery, have also been related to mental health. The existence of common green spaces also has been reported to create communication between neighbors, increase support and participation between people, and reduce the feeling of isolation among older adults (16). In the United Kingdom, a study was conducted to investigate the relationship between the size of the garden and the health of older adults, and it showed that people with larger gardens that they tended to themselves had better general health [Brindley et al., 2018, as cited in Dzhambov et al. (16)] and improved dietary intake [Davis et al., 2011; Beavers et al., 2020, as cited in Nigg et al. (18)], it may be sustained effects on healthy eating behaviors (18). Studies found that more older adults visited regional parks with large areas after COVID-19. But for small-scale parks including community parks, neighborhood parks, and paly lots, older adult visitation was negatively affected by COVID-19 (74).

In an online survey conducted in Italy, Spano et al. investigated the relationship between the presence of green spaces inside and outside the home (i.e., the presence of pots, sunlight, green landscapes, and access to private green spaces and the natural environment outside) with the level of depression, anger, anxiety, and insomnia. They found that the presence of flower pots in the house and green spaces outside the house reduced mental health problems in people during lockdown (27). During the pandemic, the green walls of buildings (especially during lockdown) played a small role in creating a sense of connection with nature and somehow increased the sense of freedom of older adults (45). In another survey conducted among 2,969 individuals in Scotland to investigate the level of psychological problems during the COVID-19 pandemic, researchers concluded that residents of urban areas, people living in deprived areas, those who do not have access to green spaces in their residential areas, and individuals who do not use green spaces outside of their homes have had more problems compared to those who live in rural areas (24).

In another study in Scotland, the effect of the following three variables was investigated: gardening in the garden, relaxing in the garden, using the garden before and after the COVID pandemic. According to the findings, older people in Scotland spent more time in the garden during the COVID-19 lockdown, and compared to before the lockdown, they reported better physical health, emotional health, mental health, and sleep quality (10).

During the COVID pandemic, the existence of small-scale green spaces has been very effective for mental activity as well as strengthening physical health, especially for older adults (10). Based on these regulations and guidelines regarding the presence of a home garden, such a space will be important for maintaining physical activity for older adults at home. Among the issues examined in long-term planning for design and construction laws are changing in the design and use of common residential spaces for residents. There are some short-term plans, such as allowing private use of open and green spaces on certain days for each family, which also had a positive impact (24). The report has shown that in the absence of a balcony or a backyard, the use of green spaces in the yard and the green spaces, especially the private garden available in the vicinity of the residence, has increased. Therefore, it is necessary to provide sufficient green space in the neighborhoods and the vicinity of the residential space (75). During the pandemic, the design of public space to provide sufficient green physical space in compliance with policies against the spread of infectious diseases has become particularly important to the effectiveness of these programs and green spaces (61).

## 4. Discussion

In our study, we have developed a conceptual framework (Figure 1) based on our research findings. To organize and categorize our findings effectively, we have classified them into three distinct categories: place-based attributes, processes, and functions. This classification allows us to better understand and analyze the various aspects related to our research topic. By considering these categories, we aim to provide a comprehensive and structured understanding of the subject matter at hand.

### 4.1. The importance of urban green spaces for older adults during the pandemic

There is a positive relationship between physical activity and the proximity, accessibility, size, quantity, and quality of urban green spaces [Coombes et al., 2010; Zhang et al., 2017; Klompmaker et al., 2018, as cited in Zwierzchowska et al. (76)]. The World Health Organization (WHO) recommends that adults engage in at least 150 min of physical activities per week to maintain physical and mental health, and during epidemics when those activities for older people are limited, home and outdoor exercises need to be replaced (35). However, unfortunately, during the COVID-19 pandemic, lockdown led to a decrease in physical activity levels among the older adult population worldwide, with important factors including an increase in sitting time and sedentary behavior, a decrease in metabolism, and a reduction in the number of walks taken and even using the green spaces.

To highlight the benefits of green spaces in older adults' lives in this narrative review, worth noting is that, during the pandemic, living in greener neighborhoods with more public green spaces can increase older people's willingness to engage in physical activities such as walking and cycling (29) as well as relaxation (65). Furthermore, individuals residing in greener areas have reported experiencing milder pandemic fatigue (29) and a lesser decline in physical activity and leisure time (50). Interestingly, there has been a significant increase in the number of individuals spending more than 2 h in these spaces after the pandemic, suggesting a preference for outdoor activities among residents. Additionally, there has been an observed rise in visits with multiple companions (65). Although increasing urban agriculture in city centers is challenging, urban farms can improve mental wellbeing by enabling residents to grow food within 100–250 m of their homes, particularly during pandemics (77). Seasonal and age-related factors have influenced the reported behavioral changes in visiting parks among older people during COVID-19. However, promoting older adults' wellbeing needs to incorporate available green networks, such as pocket parks and roof gardens, into residential neighborhoods (50). Nature-based solutions, including green infrastructure (GI), nature restoration, and forest landscape restoration, focusing on urban greening (UG), are also crucial for maintaining cities' health and wellbeing during crises. These nature-based infrastructure areas are vital for conservation and enhancement (66).

Having more benefits of green space during the pandemic depends on reducing transmission by design based on three crucial factors: the density of development and construction, the level of connectivity and design of green infrastructure, and the availability of urban green-blue spaces (48). To minimize the risk of disease transmission, it is necessary to create distance between green spaces to discourage individuals from choosing these spaces as alternative options. The selection of green spaces determines the potential for movement within the urban space network, reflecting people's inclination to use green spaces as destinations or pathways. Planners can reduce the degree of selection by designing green spaces in pedestrian-friendly areas rather than focusing solely on primary nodes and traffic links (25). Therefore, flexible policies are required to ensure access to green spaces at various scales, and monitoring exercise, either through regular visits or phone calls, can be effective in enhancing the benefits of exercising at home [Chen et al., 2021, as cited in Oliveira et al. (35)].

Furthermore, Poh et al. (78) have found that the risk of infection is lower in urban areas with forests and green spaces, while it is higher in areas with denser urbanization patterns, often indicating higher population accumulation. According to Zhang et al. (62), population density is the most significant factor in determining the risk of contamination, followed by traffic density. On the other hand, the distance from markets and commercial spaces has a lesser impact on the spread of the disease. Finally, Ye and Qiu (48) discovered that factors such as forest patches, forest edge density, and the proportion of green edges are associated with infectious disease risk, as green infrastructure, including tall trees, is critical in preventing disease transmission.

### 4.2. Effective characteristics to design green spaces for older adults during pandemics

#### 4.2.1. Socio-demographic and economic characteristics influencing the design of green spaces in pandemics

Researchers have extensively confirmed the impact of “place-oriented” factors on health outcomes [Janssen et al., 2006, as cited in Oluyomi et al. (53)]. The places where individuals reside, work, and socialize have all significantly enhanced the quality of life for older adults during the pandemic (53). This effect appears to have been amplified by various neighborhood characteristics, such as social condition, ethnicity, income, and the health status of older adults [Haan et al., 1987, as cited in Oluyomi et al. (53)], which influenced some factors such as access to care, hygiene, healthy food, recreational activities, the built environment, and neighborhood safety. Psychological resilience, age, and gender of older people have also proven to be important factors affecting the likelihood of experiencing depression and anxiety symptoms during COVID-19. Younger individuals are more susceptible to depression than older individuals, and women have experienced higher levels of psychological pressure than men (26).

Numerous social and economic factors, including age, gender, education, income, race, female-to-male population ratio, employment status, and occupation type, can influence the risk of COVID-19 (52). Individual traits such as poor health, low media literacy, advanced age, tall stature, and male gender (40) can also contribute to this risk.

Furthermore, there is a significant relationship between education level, engagement in sports, and recreational activities in urban green spaces during the epidemic. Individuals with higher education tend to recognize the importance of utilizing green spaces and improving their mental wellbeing while staying at home (77). Occupation has also been linked to exercise frequency, engagement in recreational activities, and the use of recreational spaces. For instance, individuals working in public or private jobs have the highest number of visits to green spaces (25.42%), followed by those not employed (24.82%). Monthly income is also associated with the willingness to exercise, frequency of recreational activities, and inclination to travel outside the city to access green spaces (79).

Notably, Van Tilburg et al. (38) found that elevated mental health issues, particularly emotional loneliness, are associated with individual harm, pandemic-related concerns, and decreased trust in social institutions. To promote wellbeing through addressing social distancing-related challenges among independently living older adults, the most effective interventions involve facilitating social connections, including contact with social institutions, to alleviate feelings of fear and isolation (38). A study focusing on individuals over 84 found that participants experienced an increased need for social support during this period. This positive change can be attributed to more excellent online or phone contact with family and friends, leading to a healthier life for these individuals (41).

Besides, some research indicates that older individuals in disadvantaged neighborhoods face higher social exclusion levels than in other neighborhoods. This discrepancy arises from neighborhood deprivation in areas such as access to services and amenities, social relations and civic participation, and cultural and recreational activities, as well as green spaces (40). The COVID-19 pandemic has exacerbated existing inequalities rooted in neighborhoods, with individuals of all ages in the most deprived areas of England and Wales being twice as likely to experience COVID-19 compared to those in more affluent neighborhoods [Office for National Statistics, 2022, as cited in Shoari et al. (34)]. Also, vulnerable populations, including older adults and individuals with low socio-economic status, face increased risks, as those in low-income neighborhoods also tend to have limited access to green spaces. Labib et al. (66) indicate that populations exposed to greener environments experience lower health inequities.

Moreover, the rate of COVID-19 infections is directly related to the percentage of the black population, non-native immigrants, households without access to a vehicle, and the population over 65 years old (53). The pandemic has disproportionately affected certain groups based on age, race, ethnicity, gender, disability, and sexual orientation. The spread of COVID-19 has also led to increased social stigma and discriminatory behaviors against specific ethnic backgrounds, individuals with disabilities, and marginalized groups (46). A qualitative study conducted in Mexico during COVID-19 found that women reported fear of violence as a hindrance to positive wellbeing experiences when visiting urban parks [Huerta and Cafagna, 2021, as cited in Nigg et al. (18)].

#### 4.2.2. Physical characteristic for designing green spaces in pandemics

The studies related to older adult activity show that throughout the pandemic, individuals attended parks for various activities, including sightseeing, strolling, exercising, and jogging. The purpose of such activity was to enhance their mental wellbeing, and the presence of greenery was a crucial component in attracting older adults. The landscape of parks, particularly the plants, played a significant role in this regard, as evidenced by the popularity of cherry blossoms in Olympic Forest Park, lotus flowers, and red leaves in Xishan National Forest Park (21). Incorporating seasonal activities such as flowering plants in different seasons, such as peach and cherry blossoms in the spring, lotus flowers in the summer, colorful leaf plants in the autumn, and fruit plants in the winter, was reported to enhance the appeal of these spaces. Furthermore, a significant amount of greenery in open spaces has been shown to affect the frequency of visits and overall wellbeing among older adults.

The desirability of a neighborhood for older adults is influenced by the distribution, quality, and accessibility of green spaces (79). The proximity of residential areas to urban parks and forests increases the inclination to visit these green spaces for sports or entertainment. Setiowati et al. (79) found that the frequency of visits by individuals residing within 500 meters of a park is associated with the opportunity for exercise. Still, the relationship between distances of 500 and 1,000 m has not been extensively studied. Proximity to green spaces significantly impacts the popularity and frequency of visits to these areas near residential zones (67). Restricting access to high-risk areas like playgrounds and sports facilities while allowing access to pathways and open spaces can reduce the risk of infection, promoting social distancing (34). Conversely, people's willingness to travel long distances to connect with nature in more attractive environments is evidenced by their visits to parks outside their residential areas (67).

Ye and Qiu (48) discovered a correlation between population density and the number of visits to urban green spaces. People living in areas with ample green space are more likely to utilize parks and other open green spaces, such as tree-lined streets, bike paths, or greenways. Suppose individuals allocate less time to visit green spaces in areas with limited green areas due to population density. In that case, it may lead to disparities in reducing physical activity and leisure time in the neighborhood, especially among older adults (48).

**Figure 4:**
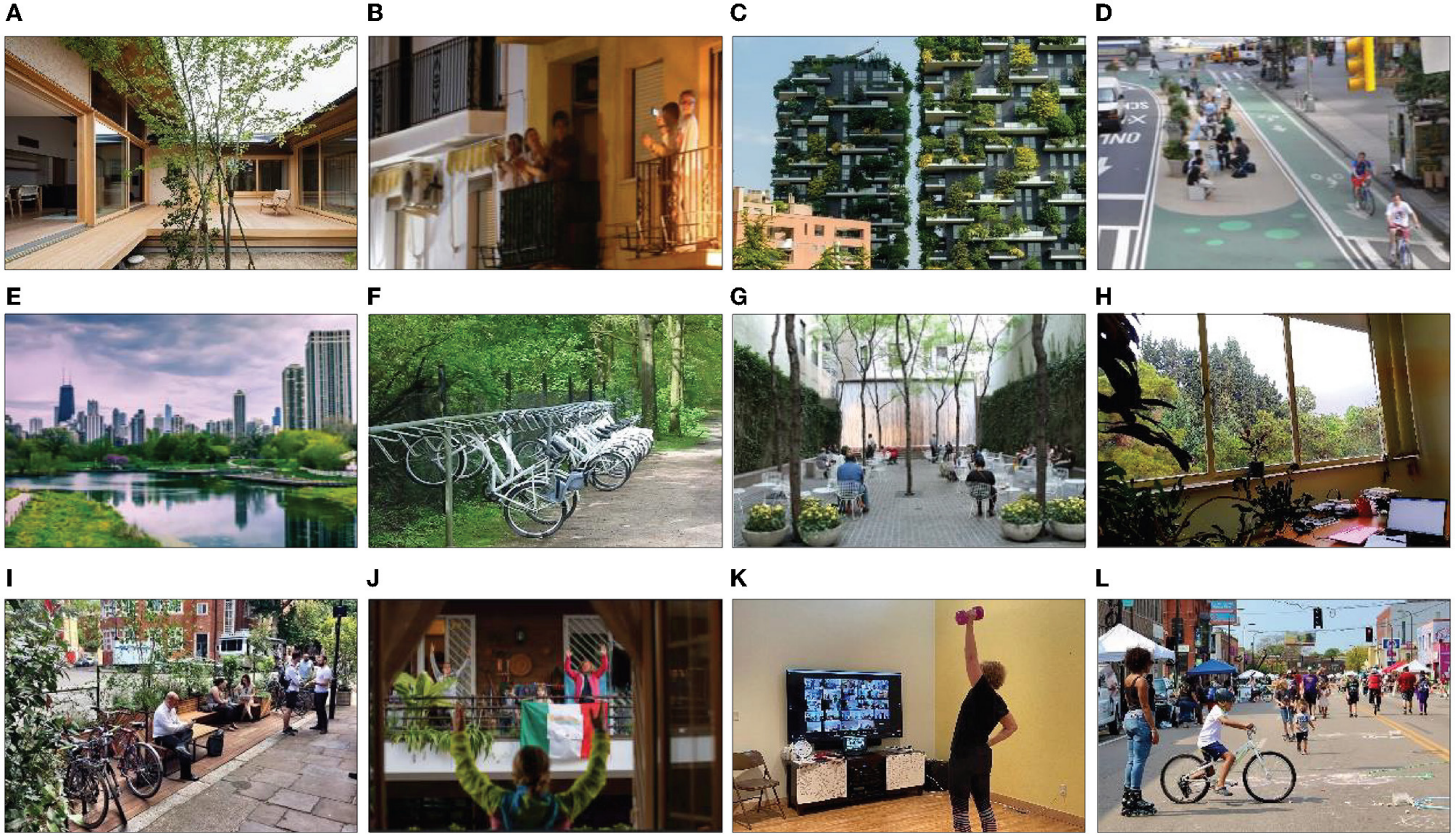
**(A–L)** are related to the design implications.

A study proposed the “Urban Meteoropathy” planning tool for high-density cities like Macau Peninsula, suggesting the implementation of multiple layers of greening on the ground, walls, and rooftops of buildings to address the lack of green spaces [Min et al., 2011, as cited in Ali et al. (30)]. To enhance social cohesion [Figure 4A; Choi and Matz-Costa, 2018, as cited in Levinger et al. (1)], safety, and security in neighborhoods, it is recommended to design houses with access to courtyards [Figure 4B; Lin et al., 2014, cited in Berdejo-Espinola et al. (20)], green spaces (Figure 4C) (66), patios, and outdoor spaces. Strengthening pedestrian and bicycle infrastructure (Figure 4D) (61) and establishing connections between green infrastructure and the urban landscape is crucial (Figure 4E) (48). Green spaces should be strategically located in walkable areas rather than primary nodes and traffic links. Effective management and maintenance of natural public spaces (66) involve considering visitor numbers, maintaining facilities, continuous monitoring, and evaluation. Sufficient funds and the collective participation of stakeholders are needed to adopt and implement nature-oriented policies. Additionally, increasing bicycle parking availability in parks and green spaces is beneficial (Figure 4F) (80).

Finally, urban planners and designers can enhance the green space experience, especially during the pandemic, by creating green walls, small parks, and gardens (Figure 4G) (66), and incorporating elements like color, plants, and sky views, and artwork to improve the indoor environment (Figure 4H) (15). Converting parking lots into parklets and using vacant lots for green spaces is also recommended [Figure 4I; (25); Hanzl, 2020; Liu and Wang, 2021; Sivak et al., 2021, as cited in Labib et al. (66)]. Implementing different mental and physical activities (Figure 4J), such as planting and maintaining plants, changing daily schedules, and adapting to conditions (22), exercise monitoring programs and offering medical consultations have proven beneficial [Figure 4K; Chen et al., 2021, cited in Oliveira et al. (35)]. During emergencies, measures like controlling entry and establishing age requirements are essential, as is organizing for vulnerable populations. Also, to increase access to parks and green spaces, the surrounding or connecting streets can be designated as open streets (Figure 4L) (80). Improving connectivity through wind corridors, high-rise buildings, and considering land shape can enhance urban green and blue spaces while influencing air conditioning. To mitigate risks in densely populated urban areas, it is necessary to separate green-blue spaces and establish dedicated wind corridors physically.

### 4.3. Future studies

Our goal was to investigate the impact of green spaces on older adults' wellbeing during the COVID19 pandemic. In the next studies, these benefits and effects can be examined in different socio-economic and cultural conditions. It is also possible to check how the policies of different countries have responded to the needs of older people during the pandemic.

## 5. Conclusion

This study draws upon available studies to identify the individual characteristics of older adults and their living environment to see the effects of the pandemic on the health of the older adult. Moreover, this study examines the qualities of green spaces, the benefits of using green spaces, the types of spaces used by older adults, and the desirable activities of the older adult in the green space during the COVID pandemic. This narrative review study highlights the substantial and enduring impact of the coronavirus on the physical and mental wellbeing of older adults. It also reveals that there is a strong relationship between the surrounding green environment and the incidence of physical problems, anxiety, and depression symptoms among the older population. In addition, in the lived experience of older adults, there was a positive encounter with the condition of the disease, which included sub-themes such as facing the crisis of acceptance and inflexibility in difficult conditions, which made the older adult adapt to the conditions related to the spread of Corona.

According to the findings, there are several stakeholders and experts, including urban planners, health professionals, local communities, and lawmakers, who may collaborate to develop successful strategies to improve people's quality of life. Officials, designers, and urban planners should consider the following measures to help reduce crises:

- Enabling access to and promoting the use of green and open spaces in various dimensions to alleviate overcrowding,
- Enhancing the quality of green spaces by improving biodiversity and tree diversity in urban areas,
- Strengthening pedestrian and bicycle paths to enhance mobility, reduce inequality, promote physical activity, and facilitate access to urban and green areas,
- Developing public and semi-public spaces to foster social interactions and cohesion within neighborhoods,
- Establishing private or shared green spaces, such as terraces and patios, to encourage activity and communication and improve the physical and mental health of the older adult,
- Enhancing neighborhood safety and security while simultaneously strengthening social capital,
- Utilizing mass media to disseminate relevant information,
- Prioritizing health measures and providing access to medical consultations, and
- Focusing on public transportation networks and their ability to minimize risks and injuries, especially for the older adult.

As the global population continues to age, there is an urgent need for further research in the field of the physical and mental health of older adults. This is particularly crucial in the context of urban environments, where the majority of the population resides, and where environmental factors can significantly impact health outcomes. To effectively address the challenges facing the older adult population, it is critical to understand the influence of various environmental variables on their physical and mental health.

## Author contributions

All authors listed have made a substantial, direct, and intellectual contribution to the work and approved it for publication.
