# Supplementary material for: Green space and the health of the older adult during pandemics: a narrative review on the experience of COVID-19
Source: Front Public Health. 2023 Jul 31;11:1218091. doi: 10.3389/fpubh.2023.1218091 (PMC10433209; doi:10.3389/fpubh.2023.1218091)
Supplement: Supplementary file 1 [file Table_1.docx]

**Appendices**

**Table 1. Reviewed Articles (Source: Authors)**

| **Participates** | **Type** | **Location** | **Year** | **Article** | **NO** |
| --- | --- | --- | --- | --- | --- |
| ـــ | Review Article | Brazil, Spain | 2020 | ([Sánchez-González et al., 2020](#Sánchez_2020)) | 1 |
| 5218 Participates | ـــ | Spain, UK, US, Germany, France, Portugal, Italy, New Zealand, Mexico | 2020 | (Pouso et al., 2021) | 3 |
| 786 Neighborhood | ـــ | Harris County, Texas,USA | 2021 | ([Oluyomi et al., 2021](#Oluyomi_2021)) | 4 |
| ـــ | ـــ | UK | 2020 | ([Buffel et al., 2020](#Buffel_2020)) | 5 |
| ـــ | ـــ | ـــ | 2020 | (Ugolini et al., 2020) | 6 |
| ـــ | ـــ | Guangzhou, China | 2021 | (Li et al., 2021) | 7 |
| 809 Participates -75 Years Old, 80 Years Old or 85 Years Old | ـــ | Finland | 2021 | ([Rantanen et al., 2021](#Rantanen_2021)) | 8 |
| 528 Participates -People over 65 in Spain  The studied group was mainly female, with an average age of 69 years and a high level of education | ـــ | Spain | 2020 | ([Rodríguez-González et al., 2020](#Rodríguez_2020)) | 9 |
|  | ـــ | Brazil |  | ([Mello et al., 2022](#Mello_2022)) | 10 |
| 1679 participates  Internet questionnaire of people over 65 years old | ـــ | Netherland, Switzerland | 2020 | ([Van Tilburg et al., 2020](#van_2020)) | 11 |
| ـــ | ـــ | India, UAE | 2021 | ([Sharma & Sharma, 2021](#Sharma_2021)) | 12 |
| ـــ | ـــ | Uk | 2021 | ([Geary et al., 2021](#Geary_2021)) | 13 |
| ـــ | ـــ | Wuhan, China | 2021 | (Ye & Qiu, 2021a) | 14 |
| Two Neighborhood -661 Participates | ـــ | Hong Kong, China | 2021 | (Y. Yang et al., 2021) | 15 |
| All Age Groups | Quantitative | Newyork, USA, France | 2020 | (Whittle & Diaz-Artiles, 2020b) | 16 |
| Two studies were conducted: In the first study, 2969 people participated, with an average age of 54  In the second study, 502 people participated, the average age was 53 | ـــ | Scotland | 2021 | ([Hubbard et al., 2021](#Hubbard_2021)) | 17 |
| 3866 participates | ـــ | Italy, Hong Kong | 2021 | ([Spano et al., 2021](#Spano_2021)) | 18 |
| 171 participatesover 84 years old | ـــ | Scotland | 2020 | (Corley et al., 2021a) | 19 |
| 1002 participats | ـــ | Australia, Colombia | 2021 | (Berdejo-Espinola et al., 2021) | 20 |
| 395 participates | ـــ | Italy | 2021 | ([Bartalucci et al., 2021](#Bartalucci_2021)) | 21 |
| 137 participants over 84 years old | ـــ | Scotland | 2021 | ([Okely et al., 2021](#Okely_2021)) | 22 |
| 214 neighborhood | ـــ | Chicago , USA | 2021 | (Zhang et al., 2021a) | 23 |
| A 54-year-old public transport worker | ـــ | Coroatia | 2021 | ([Žaja et al., 2021](#Žaja_2021)) | 25 |
| 411 participates | ـــ | Saudi Arabia | 2020 | ([Zubair et al., 2020](#Zubair_2020)) | 26 |
|  | ـــ | Netherlands, US |  | ([Labib et al., 2022](#Labib_2022_a)) | 27 |
| Over 60 years old | ـــ | New Zealand | 2021 | ([Humberstone, 2021](#Humberstone_2021)) | 28 |
| Over 50 years old | ـــ | Brazil | 2020 | (Roschel et al., 2020b) | 29 |
| people over 18 years old living in Shiraz metropolis  1265 participates (386 tons living in the city center and 879 tons not living in the city center) | ـــ | Shiraz, Iran | 2021 | ([Shaer et al., 2021](#Shaer_2023)) | 30 |
| 144 participates | ـــ | Newyork, USA | 2021 | ([Tribby & Hartmann, 2021](#Tribby_2021)) | 31 |
| 19 participates over | Qualitative | Uk, Irland | 2020 | ([Brooke & Clark, 2020](#Brooke_2020)) | 32 |
| 18817 steps counts | ـــ | Yokohama,Japan | 2021 | ([Hino & Asami, 2021](#Hino_2021)) | 33 |
| Over 65 years old participates | Quantitative | Milan,Italy | 2021 | ([Guida & Carpentieri, 2020](#Guida_2020)) | 34 |
| - | ـــ | Wuhan, China | 2020 | ([Zhang et al., 2020](#Zhang_2020)) | 35 |
| ـــ | Review Artcile | Australia | 2021 | ([Levinger et al., 2021](#Levinger_2021)) | 36 |
| ـــ | Review Artcile | China | 2021 | (Zhu & Xu, 2021) | 37 |
| ـــ | ـــ | UK | 2021 | (Pan et al., 2021) | 38 |
| - | - | 10 capital cities in Europe (Amsterdam, Brussels, Berlin, Copenhagen, Dublin, Lisbon, London, Madrid, Paris and Rome) | 2020 | (Carpentieri et al., 2020) | 39 |
| - | - | Atlanta | 2022 | (Weiying et al., 2022) | 40 |
